# *Plasmodium* spp. membrane glutathione S-transferases: detoxification units and drug targets

**DOI:** 10.15698/mic2014.11.177

**Published:** 2014-10-23

**Authors:** Andreas M. Lisewski

**Affiliations:** 1 Department of Molecular and Human Genetics, Computational and Integrative Biomedical Research Center, Baylor College of Medicine, Houston, TX 77030, USA.

**Keywords:** Plasmodium, malaria, detoxification, glutathione, artemisinin

## Abstract

Membrane glutathione S-transferases from the class of membrane-associated proteins in eicosanoid and glutathione metabolism (MAPEG) form a superfamily of detoxification enzymes that catalyze the conjugation of reduced glutathione (GSH) to a broad spectrum of xenobiotics and hydrophobic electrophiles. Evolutionarily unrelated to the cytosolic glutathione S-transferases, they are found across bacterial and eukaryotic domains, for example in mammals, plants, fungi and bacteria in which significant levels of glutathione are maintained. Species of genus *Plasmodium*, the unicellular protozoa that are commonly known as malaria parasites, do actively support glutathione homeostasis and maintain its metabolism throughout their complex parasitic life cycle. In humans and in other mammals, the asexual intraerythrocytic stage of malaria, when the parasite feeds on hemoglobin, grows and eventually asexually replicates inside infected red blood cells (RBCs), is directly associated with host disease symptoms and during this critical stage GSH protects the host RBC and the parasite against oxidative stress from parasite-induced hemoglobin catabolism. In line with these observations, several GSH-dependent *Plasmodium* enzymes have been characterized including glutathione reductases, thioredoxins, glyoxalases, glutaredoxins and glutathione S-transferases (GSTs); furthermore, GSH itself have been found to associate spontaneously and to degrade free heme and its hydroxide, hematin, which are the main cytotoxic byproducts of hemoglobin catabolism. However, despite the apparent importance of glutathione metabolism for the parasite, no membrane associated glutathione S-transferases of genus *Plasmodium* have been previously described. We recently reported the first examples of MAPEG members among *Plasmodium *spp.

One reason why *Plasmodium* MAPEG enzymes might have been missed in the past is the fact that there is no significant sequence homology between known MAPEGs and *Plasmodium* protein sequences. This is not unexpected given that the genome sequence of the human malaria parasite *Plasmodium falciparum, *the most studied *Plasmodium *species and the causative agent of the most severe form of malaria in humans, still lacks functional annotations for most of its genes. To address this problem, we developed a network-based functional prediction method with increased sensitivity over traditional sequence homology based methods (termed *graph-based information diffusion on compressed supergenomic networks*) that integrates evolutionary and interaction-specific links over hundreds of genomes. Among many produced predictions, the method pointed to the small parasitophorous vacuolar membrane antigen *Pf*EXP1 (*Plasmodium falciparum* exported protein 1) as having GST activity. This possibility warranted further investigation because *Pfexp*1 was already known as one of the most highly transcribed genes during ring and trophozoite stages of *falciparum* malaria, and because it occupies a locus which has resisted gene disruption attempts and thus indicated its essentiality.

We confirmed the computational prediction experimentally: after heterologous expression in *E. coli* GST activity of purified *Pf*EXP1 toward the standard substrate 1-chloro-2,4-dinitrobenzene (CDNB) was measured. The link to malaria parasite biology was further strengthened by our next observation that *Pf*EXP1 as well as its close ortholog *Plasmodium yoelii* *Py*HEP17 (17 kDa hepatocyte protein) both use hematin as a substrate, i.e. they conjugate reduced glutathione onto hematin to form hematin-GSH adducts. Thus far, these enzymes appear to be the only known MAPEG GSTs with hematin substrate specificity (Figure 1) and may therefore enable the parasite to buffer oxidative stress produced through excess hematin. This protective activity may be important during the ring stages of the parasite, when *Pf*EXP1 levels are high, and when hemoglobin catabolism has already started but when significant hematin biocrystals (hemozoin) buildup and the formation of a main food vacuole have yet to begin. Interestingly, these ring stages have been also described as highly sensitive to artemisinins, the family of sesquiterpene lactones that contain a peroxide bridge, and which today form the frontline treatment against malaria. Because of the large body of evidence that artemisinins require a hemoglobin degradation product, possibly heme or hematin, as the molecular activator of their peroxide bridge and thus of their full antimalarial action, we hypothesized that artemisinins might inhibit the membrane GST activity of *Pf*EXP1 in a hematin dependent manner. *In vitro* inhibition studies confirmed that in the presence of free hematin artesunate (a water soluble artemisinin derivative) is a potent and competitive inhibitor with a half maximal inhibitory concentration near 1 nM. Without hematin, artesunate inhibition of *Pf*EXP1 GST activity toward CDNB was 100-fold less potent and uncompetitive. This strong hematin dependence may explain why *Pf*EXP1, which is also expressed during host liver stages when parasite induced hemoglobin degradation does not yet take place, would be targeted by artemisinins specifically after RBC invasion.

**Figure 1:**
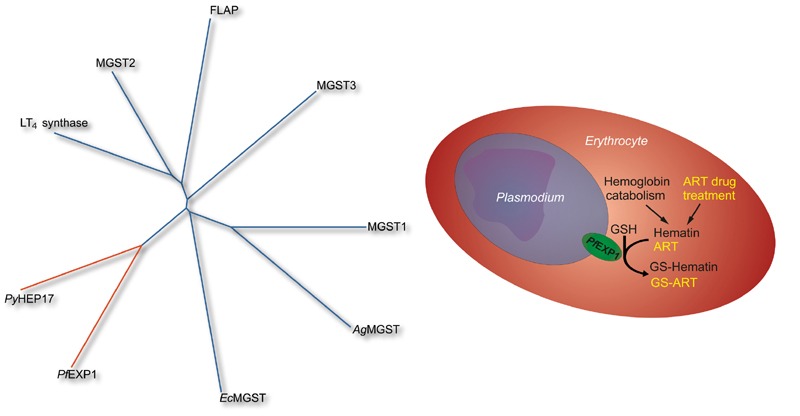
FIGURE 1: (Left) Representative dendrogram of the MAPEG superfamily including the malaria parasite GSTs *Plasmodium yoelii* HEP17 (*Py*HEP17) and *Plasmodium falciparum* EXP1 (*Pf*EXP1), *Escherichia coli* MGST (*Ec*MGST), *Anopheles gambiae *MGST (*Ag*MGST), rat MGST1, and human MGST3, 5-lipoxygenase activating protein (FLAP), MGST2, and leukotriene C4 synthase. (Right) Proposed model of *Pf*EXP1 in *Plasmodium falciparum* infected erythrocytes. The membrane protein *Pf*EXP1 catalyzes the conjugation of reduced glutathione (GSH) onto hematin and onto the drug artemisinin (ART) and thus contributes to their detoxification.

Competitive inhibition of *Pf*EXP1 through artesunate opened the possibility that *Pf*EXP1 might play a role in the metabolic degradation of artesunate, and we observed that *Pf*EXP1 indeed facilitates the conjugation of GSH with artesunate (Figure 1) both *in vitro* and in parasites cultured under selective pressure through dihydroartemisinin. These selected parasites, which recrudesce earlier and with a larger population after exposure to high levels of artemisinins than their drug-sensitive parental strains, show differential expression of *Pfexp*1 at mRNA and at protein levels. In contrast to the membrane bound *Pf*EXP1, the cytosolic *Pf*GST lacks this catalytic ability, is not differentially expressed in these more resistant strains, and thus is probably incapable to modulate parasite susceptibility to artemisinins. Interestingly, other common antimalarial drugs such as chloroquine and atovaquone are not catalytically conjugated onto GSH through *Pf*EXP1 (our unpublished data.) Thus *Pf*EXP1 appears to be involved in a metabolic degradation pathway specific to artemisinins which can be upregulated in drug pressured parasites that are less sensitive to artemisinin. These results suggest that membrane associated GSTs in *Plasmodium* spp. are relevant to the recently observed loss of artemisinin efficacy in field and clinical studies. This loss has been primarily evidenced through longer times it takes to clear parasites from patient blood, and *Plasmodium* membrane GSTs may constitute a significant part of a parasite defense system to withstand exposure to artemisinins.

